# Isolation and Differentiation of Neurons and Glial Cells from Olfactory Epithelium in Living Subjects

**DOI:** 10.1007/s12035-023-03363-2

**Published:** 2023-04-28

**Authors:** Paula Unzueta-Larrinaga, Rocío Barrena-Barbadillo, Inés Ibarra-Lecue, Igor Horrillo, Aitor Villate, Maria Recio, J. Javier Meana, Rebeca Diez-Alarcia, Oihane Mentxaka, Rafael Segarra, Nestor Etxebarria, Luis F. Callado, Leyre Urigüen

**Affiliations:** 1 Department of Pharmacology, University of the Basque Country, UPV/EHU, Leioa, Spain; 2 Biocruces Bizkaia Health Research Institute, Barakaldo, Spain; 3 Department of Nursery, University of the Basque Country UPV/EHU, Leioa, Spain; 4 Centro de Investigación Biomédica en Red de Salud Mental, CIBERSAM, Madrid, Spain; 5 Department of Analytical Chemistry, University of the Basque Country UPV/EHU, Leioa, Spain; 6 PiE-UPV/EHU, Plentzia, ItsasEstazioa, Areatza Pasealekua, 48620 Plentzia, Spain; 7 Department of Psychiatry, Cruces University Hospital, Barakaldo, Spain; 8 Department of Neurosciences, University of the Basque Country, UPV/EHU, Leioa, Spain

**Keywords:** Olfactory epithelium, Neurospheres, Neurons, Glia, Neuropsychiatric diseases, PSA-NCAM

## Abstract

The study of psychiatric and neurological diseases requires the substrate in which the disorders occur, that is, the nervous tissue. Currently, several types of human bio-specimens are being used for research, including postmortem brains, cerebrospinal fluid, induced pluripotent stem (iPS) cells, and induced neuronal (iN) cells. However, these samples are far from providing a useful predictive, diagnostic, or prognostic biomarker. The olfactory epithelium is a region close to the brain that has received increased interest as a research tool for the study of brain mechanisms in complex neuropsychiatric and neurological diseases. The olfactory sensory neurons are replaced by neurogenesis throughout adult life from stem cells on the basement membrane. These stem cells are multipotent and can be propagated in neurospheres, proliferated in vitro and differentiated into multiple cell types including neurons and glia. For all these reasons, olfactory epithelium provides a unique resource for investigating neuronal molecular markers of neuropsychiatric and neurological diseases. Here, we describe the isolation and culture of human differentiated neurons and glial cells from olfactory epithelium of living subjects by an easy and non-invasive exfoliation method that may serve as a useful tool for the research in brain diseases.

**Supplementary Information:**

The online version contains supplementary material available at 10.1007/s12035-023-03363-2.

## Introduction

Modeling neuropsychiatric disorders is extremely challenging given the subjective nature of many of the symptoms and the lack of biomarkers and objective diagnostic tests [1]. Currently, several types of human bio-specimens are being used for research, including postmortem tissue, cerebrospinal fluid, induced pluripotent stem (iPS) cells, or induced neuronal (iN) cells [2–4].

The human olfactory epithelium supplies information to the olfactory bulbs of the brain and displays a continuous and powerful neurogenesis in humans, by which olfactory sensory neurons are replaced [5]. Neuronal progenitor cells present in the olfactory epithelium are defined as undifferentiated clonogenic cells that possess the capacity for self-renewal, to generate neuronal or astrocytic cells, as well as to remain as neurospheres in cell culture [6–8]. In this context, the olfactory epithelium has received increased interest as a window to brain mechanisms in complex psychiatric diseases [4, 9–13]. To date, other studies have developed different methods for obtaining the olfactory neuroepithelium (ON) sample, invasively in biopsies after nasal surgery [14], postmortem at autopsies [15], or even by a recent non-invasive method [16]. However, all these studies have mainly focused on the evaluation of the olfactory epithelium cells, without further differentiation, that could provide more information on the disease-specific alterations in neurons and/or glial cells.

We aimed to obtain and grow the neural progenitor cells (NPCs) from the olfactory epithelium and differentiate into neurons or glial cells for obtaining specific cell-type cultures in sufficient quantity for the study of neuropsychiatric disorders.

The final objective of this work is the development of a non-invasive, easy, reproducible, and reliable method for the isolation, culture, and characterization of neurons and glial cells from human olfactory epithelium. In this way, a unique resource can be obtained for the investigation of the neuronal and glia-related molecular mechanisms underlying psychiatric and neurological disorders.

## Material and Methods

**Fig. 1:**
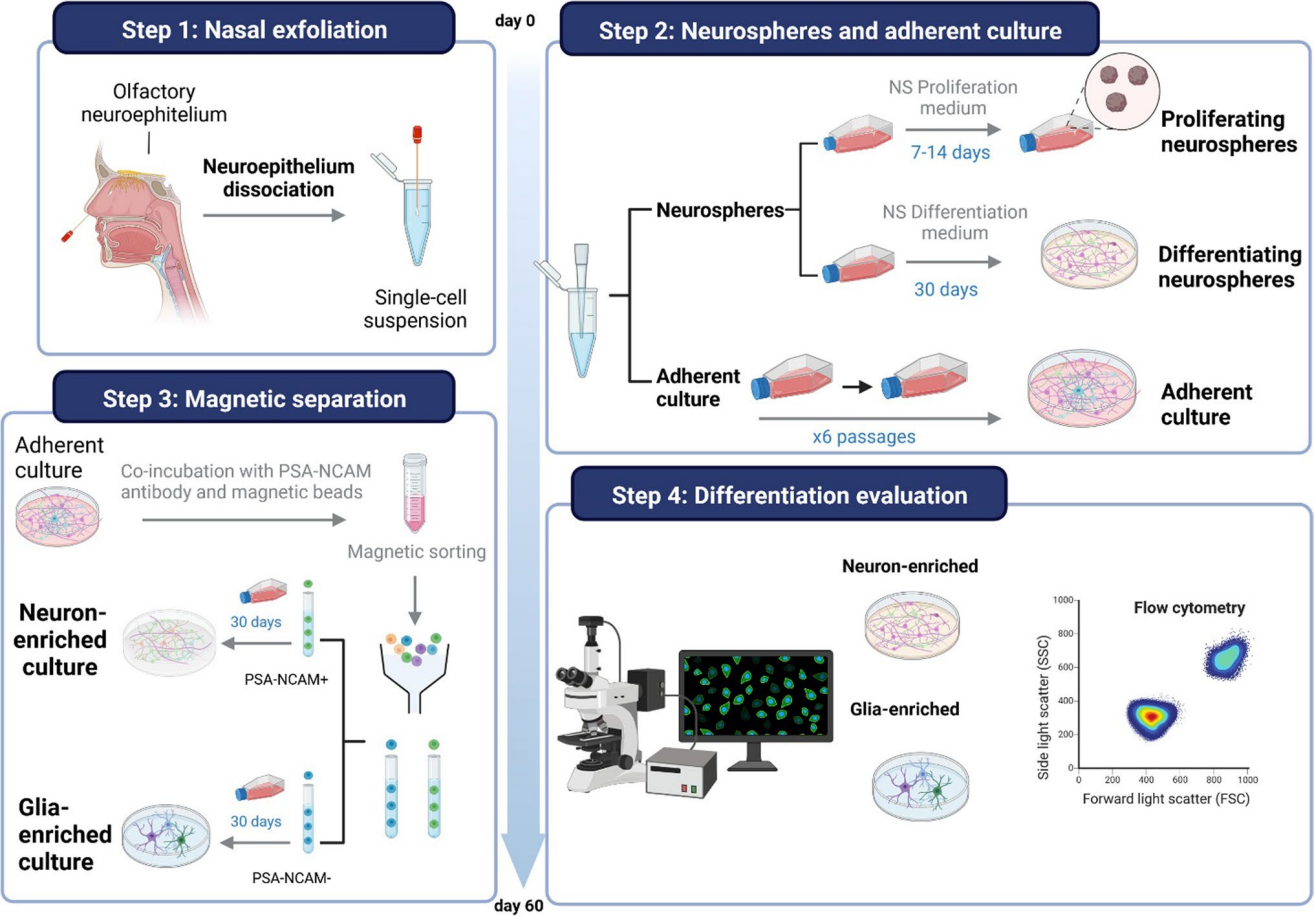
Workflow of the complete step-by-step protocol for differentiating neurons and glial cells from olfactory epithelium

A complete workflow of the method is described in Fig. 1.

### Nasal Exfoliation

The olfactory epithelium was obtained by nasal exfoliation from eight healthy donors as previously described [16]. Briefly, the nasal area was exfoliated by circular movements with a sterile swab (brush 2.4 cm long and 3 mm in diameter) introduced into the nasal cavity. Four different swabs were used for each donor for exfoliating the middle and upper regions of the two nasal cavities. After exfoliation, each brush was placed in tubes with 250 µl conventional culture medium (Dulbecco’s Modified Eagle Medium/Ham F-12 (DMEM/F12) supplemented with 10% fetal bovine serum (FBS), 2% glutamine, and 1% streptomycin-penicillin (supplemented DMEM medium) (Gibco BRL, USA), keeping the samples on ice. Four samples were obtained for each subject, two (middle and upper regions) for each nostril. The sample from the upper part of the right nostril was mixed with the sample from the middle part of the left nostril and put together in the same tube; the same was done with the other two samples. One of these mixed samples was used for the culture of adherent cells and the other for the culture of neurospheres.

Neurospheres were cultured and characterized for the demonstration of the presence of stem cells in the olfactory epithelium. Obtaining differentiated cells from neurospheres is extremely complicated, and the number of cells obtained is very low. For this reason, the generation of neuron- or glia-enriched cultures was performed from adherent cultures.

### Culture of Neurospheres from Olfactory Epithelium

For the culture of these neurospheres, the samples previously obtained by nasal exfoliation were undergone to a manual disintegration followed by centrifugation (500 g for 5 min, room temperature (RT)). The supernatant was removed and the pellet was resuspended in 500 µl of NeuroCult™ NS-A Proliferation Medium (StemCell Technologies, France) supplemented with 20 ng/ml human recombinant EGF, 10 ng/ml human recombinant bFGF, and 2 μg/ml heparin solution (supplemented NeuroCult™ NS-A Proliferation Medium) and seeded in a 25 cm^2^ flask with 4 ml of this supplemented medium. Cells were grown at 37 °C with 5% CO_2_ and observed under a direct bright-light microscope (Primovert KMAT, Zeiss, Germany). Neurospheres were then submitted to passages to test their regeneration and multiplication capacity. In this case, the passage was done when neurospheres reach approximately 100–150 µm in diameter, usually 7–14 days after seeding, although in some cases, it may take up to 21 days. If the medium became acidic before the neurospheres reached 100–150 µm in diameter, 500/1000 µl of supplemented proliferation medium was added to renew nutrients and prevent cell death. On passage 5, neurospheres were isolated by centrifugation and fixed for immunocytochemistry characterization.

On the other hand, another flask of neurospheres was maintained without making any passes. Only additions of supplemented proliferation medium were made for renewing of nutrients every 2 days until adhesion to the flask spontaneously occurred. When neurospheres were adhered to the flask, the neurosphere culture medium was changed to NeuroCult™ NS-A Differentiation medium (StemCell Technologies, France) to promote the differentiation of the cells. This would result in a pure culture of cells of neuronal lineage. After 30 days in differentiation medium, the mix of cells was fixed for immunocytochemistry.

### Adherent Culture of Cells from Olfactory Epithelium

For the culture of adherent cells, the samples were manually disaggregated and seeded in a 25 cm^2^ flask and cells were grown at 37 °C and with 5% CO_2_ in supplemented DMEM medium. When the culture reached confluence, cells were washed twice with sterile and tempered phosphate-buffered saline (PBS 1X), detached with 0.5% trypsin–EDTA (GibcoBRL, USA), neutralized with supplemented DMEM medium, and centrifuged at 1000 g RT for 5 min and the pellet was resuspended in supplemented DMEM medium. Finally, cells were seeded on a larger surface to obtain a stock of cells. In this way, cells at different stages of maturation were obtained. Characterization of cells with immunocytochemistry was carried out on cells cultured to passage 5.

### Neuron and Glia Culture Enrichment Using Magnetic Activated Cell Sorting

For the isolation and purification of neuron- or glia-enriched cultures, anti-PSA-NCAM micro beads (Miltenyi Biotec, Germany) were used for the positive selection of PSA-NCAM^+^ cells from the adherent cell cultures. Anti-PSA-NCAM micro beads recognize polysialic acid (PSA), which is linked to the extracellular domain of the neural cell adhesion molecule (NCAM, CD56) [17]. Once culture reached confluence at passage 5 (approximately 4/5 days after seeding, although it depends on the cells in the culture), cells were washed twice with sterile and tempered PBS 1X, detached with 0.5% trypsin–EDTA, neutralized with differentiation medium, and centrifuged at 300 g for 10 min. The supernatant was removed and the pellet was resuspended in 60 µl of buffer (0.5% bovine serum albumin (BSA) in PBS 1X) per 10^7^ total cells (Fluidlab R-300, Anvajo, Germany) well mixed and incubated for 10 min RT at 4 °C. The cells were then incubated with 20 µl of anti-PSA-NCAM MicroBeads/10^7^ cells for 15 min RT at 4 °C. Finally, cells were washed by adding 1 ml of buffer/10^7^ cells and centrifuged at 300 g RT for 10 min, and the pellet was resuspended in 500 µl of buffer/10^8^ cells.

After magnetic labeling, cells were passed through a magnetic activated cell sorting (MACS) column (Miltenyi Biotec, Germany) placed in a strong permanent magnet. The ferromagnetic spheres in the column amplify the magnetic field by 10,000-fold, thus inducing a high gradient. Unlabeled cells (PSA-NCAM^−^) pass through the column while magnetically labeled cells (PSA-NCAM^+^) are retained within it. After removal of the column from the magnetic field, the retained fraction was eluted. Both fractions, labeled (PSA-NCAM^+^, neuron-enriched fraction) and non-labeled (PSA-NCAM^−^, glia-enriched fraction), were completely recovered. The glia-enriched culture was then seeded in NeuroCult™ NS-A Differentiation medium with medium changes every 2 days for approximately 30 days. The neuron-enriched culture, however, was first seeded in supplemented DMEM/F12 + medium to allow cell growth, and after 7 days, the medium was changed to NeuroCult™ NS-A Differentiation with medium changes every 2 days until 30 days, when cells were characterized by immunocytochemistry and flow cytometry.

### Characterization of Cultures by Immunocytochemistry

After the MACS, approximately 5 days are needed for the culture to stabilize; the cells that survive the magnetic separation begin to adhere to the flask and to differentiate. At this time, we performed the PSA-NCAM immunocytochemistry in both types of cultures, to ensure the quality of the separation. Afterwards, we let the cultures grow for approximately 30 days, and the immunocytochemical characterization of the neuron-enriched and glia-enriched cultures was carried out. Cells were washed twice with sterile and tempered PBS 1X and fixed with 4% paraformaldehyde (PFA; Sigma-Aldrich Chemicals, Spain) in PBS for 20 min at 4 °C and shaking. Cells were washed again with PBS 1X, then 2 times with A solution (PBS 1% BSA + 0.02% azide) at RT for 5 min and with agitation at 90 rpm, continued by a quick wash with B solution (PBS 0.5% Triton X100) at RT for 5 min and with agitation at 90 rpm and two final washes with A solution again (RT for 5 min and with agitation at 90 rpm). After washing, cells were incubated for 1 h RT and agitation at 90 rpm with blocking solution (PBS 2% BSA + 0.02% azide). Finally, cells were then incubated overnight at 4 °C with the primary antibodies diluted in a solution with PBS 0.5% BSA and 0.1% azide.

Antibodies against Nestin, Sox2, and Musashi-1 were used for staining the neurospheres; PSA-NCAM, βIII-Tubulin, Neuronal-Nuclei (NeuN), Nestin, Microtubule-associated protein 2 (MAP2) and Microtubule-associated protein 1B (MAP1B) for neural cells; glial fibrillary acidic protein (GFAP) for astrocytes; and epithelial cell adhesion molecule (EpCAM) for epithelial cells. A detailed description of primary antibodies and dilutions can be found in Supplemental Table 1.

In the next day, the primary antibodies were removed and washed three times with solution A (RT, 5 min; agitation, 90 rpm). Finally, cells were incubated with the secondary antibodies (90 min, RT; agitation, 90 rpm; and darkness) (Supplemental Table 2). Secondary antibodies were removed, and cells were washed again three times with solution A (RT, 5 min; agitation, 90 rpm). The nuclei were stained with 4′, 6′-diamidino-2-phenylindole dihydrochloride (DAPI 1/5000; Panreac Applichem (A4099), Spain). The immunocytochemistry of the neurospheres was carried out in the same way but with a previous centrifugation for the precipitation of the neurospheres on a slide. Images were acquired with a Nikon Eclipse 80I and processed with ImageJ software.

### Characterization of Neuron- and Glia-Enriched Cultures with Flow Cytometry

#### Sample Preparation

The flow cytometry assays were performed 30 days after the MACS. During this 30-day period, cells are allowed to grow and differentiate with the final objective of having a sufficient amount of differentiated cells.

Cells were washed twice with sterile and tempered PBS 1X, 0.5% trypsin–EDTA was added, and cells were placed in the incubator (37℃) for 2 min. When cells were completely detached, the trypsin was neutralized by pipetting with differentiation medium up and down over the surface of the flask to detach as many cells as possible. Once it was verified that the cells were correctly detached, the cell suspension was collected in a falcon. Cells were then centrifuged at 500 g for 10 min, the supernatant was discarded, and the pellet was resuspended in 600 µl of blocking solution (1 h, with agitation at 4℃). Primary antibodies were added over the blocking solution overnight with agitation at 4℃. In the following day, the cell suspension was centrifuged at 500 g for 10 min, the supernatant was discarded, and the pellet was resuspended in 600 µl of sterile (cold) PBS 1X. Another centrifugation was made at 500 g for 10 min, the supernatant was discarded, and the pellet was resuspended in 600 µl secondary antibody dilution in blocking solution. After incubating 90 min with agitation at 4℃, cells were washed and centrifuged twice at 500 g for 10 min. The resulting pellet was then resuspended in 600 µl of PBS 1X the first time and the second time in 600 µl of HBSS solution (Hank’s balanced salt solution) which is the solution needed to analyze the sample in the cytometer.

#### Fluorescence-Activated Cell Sorting (FACS)

The samples were analyzed and sorted in a BD FACSJazz Cell sorter equipped with two independently aligned B488 and Y/G561 lasers. System pressure is 27 psi. Prior to analysis, the sample was filtered through a sterile sieve with a pore diameter of 50 µm (BD Filcon, sterile, cup-type, ref: 340,629) to avoid clumps in the sample line.

### Quantification of Prostaglandin E2, Interleukin-6, Ratio of Kynunerine/Tryptophan, and Interferon Gamma in Culture Medium

Prostaglandin E2 (PGE2), interleukin-6 (IL6), ratio of kynunerine (Kyn)/tryptophan (Trp), and interferon gamma (IFNγ) were quantified with commercial enzyme-linked immunosorbent assay (ELISA) kits (human IFNγ ELISA Kit, cat. no. BMS228, Invitrogen, Thermo Fisher Scientific, Inc.; human IL-6 ELISA kit, cat. no. BMS213-2, Invitrogen, Thermo Fisher Scientific, Inc.; and Prostaglandin E 2 ELISA Kit—monoclonal, cat. no. 514010, Cayman Chemical, Ann Arbor, MI, USA) in culture medium samples, according to the manufacturer’s instructions. No dilution was performed, and the absorption peak was at 450 nm. Protein content in culture medium was measured by Bradfords’ method [18], and results are shown as pg/mg protein of culture medium.

HPLC with electrochemical detection was used to quantify Trp and Kyn in culture medium samples. Each sample was first diluted 1:5 in 0.1 M HClO_4_ and 100 µl EDTA and filtered by centrifugation at 18,000 g × 15 min using Costar® Spin-X® Centrifuge Tubes (0.22 µm Pore CA Membrane, Corning). The concentration of Trp and Kyn was estimated with reference to standards prepared and injected on the same day. The mobile phase composition was 150 mM H_2_NaPO_4_, 0.2 mM EDTA, 4.3 mM octyl sodium sulfate (pH 6.3), and 8% (vol/vol) methanol. The mobile phase was filtered and delivered at a flow rate of 0.2 ml/min by a Hewlett-Packard model 1200 pump. Separation was carried out at 30 °C on a Zorbax Eclipse Plus column (3.5 μm C18, 2.1 × 150 mm, Agilent Technologies, Spain). Amperometric detection of Trp and Kyn was done by a Hewlett-Packard model 1049 A detector at an oxidizing potential of + 950 mV. Results are expressed as Kyn/Trp concentration ratio.

### Ethical Aspects

All subjects (*n* = 8) were volunteers without a psychiatric condition and gave their written consent before sample extraction. The entire procedure was approved by the corresponding ethics committee (CEI E22/27).

## Results and Discussion

### Olfactory Epithelium Cells from Living Subjects Are Able to Form Growing and Proliferating Neurospheres

NPCs in the olfactory epithelium are responsible for renewing the population of sensory neurons throughout life, maintaining a lifelong capacity of regeneration after injuries [19, 20]. When the in vitro culture conditions are adequate, the isolated progenitor neural cells, with high mitotic capacity, are capable of growing in the form of spherical aggregates, called neurospheres. This is an inherent capacity of NPCs [21]. These structures derived from the olfactory mucosa might give rise to neurons and glia. So, they are very interesting for establishing new human models for the study of diseases that affect the central nervous system. As they do not require genetic reprogramming, they are very useful for understanding aspects of the etiology of the disease, improving diagnosis, monitoring treatment, or promoting the development of new therapeutic drugs [6].

**Fig. 2:**
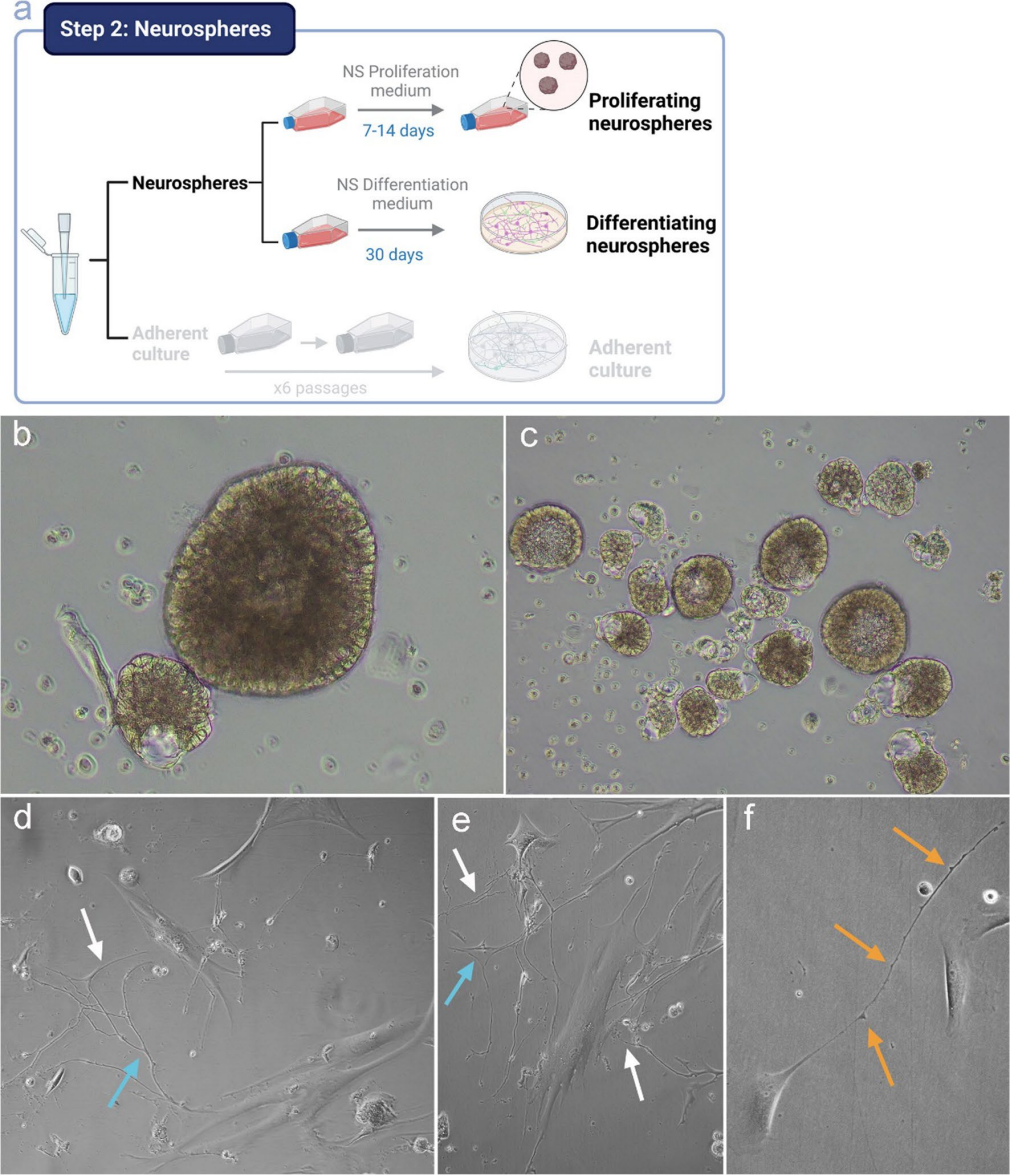
Neurosphere cultures. Schematic representation of the workflow for the culture of neurospheres (**a**). Bright-field visual characterization (10X) of growing neurospheres (**b**) and proliferating neurospheres (**c**). Cell with neuronal shape and long prolongation (white arrow) connecting with another similar cell (blue arrow) including dendritic spines (orange arrow) (**d**, **e**, **f**)

To demonstrate the presence and functionality of NPCs after nasal exfoliation in our control subjects, we set out to obtain and multiply cultured neurospheres (Fig. 2a). As seen in Fig. 2, when NPCs were cultured in a specific proliferation medium, cells formed rounded aggregates (Fig. 2b). These neurospheres, being formed by multipotent stem cells, have the ability to divide without differentiation (Fig. 2c). When the neurospheres were disaggregated by centrifugation and afterwards with manual pipetting disaggregation and cultured again in a new flask, these independent cells reassembled, giving rise to the formation of new neurospheres for unlimited proliferation, a remarkable characteristic of neurospheres [22–26]. When neurospheres were adhered to the flask, after 10/12 days of culture in differentiation medium, cells begin to appear in different stages of differentiation. As can be seen in Fig. 2d, e, and f, some of these cells have neuronal profile, with extensions that connect with other cells, and some have even developed synaptic buttons.

The ability to obtain growing and dividing neurospheres and that these neurospheres were able to differentiate to neural-like cells demonstrates that NPCs can be isolated from nasal exfoliation in living subjects.

### Olfactory Epithelium-Derived Neurospheres Express NPC-Specific Markers

Although, to date, no specific marker for neurospheres has been described, these formations transiently express the protein Nestin [27]. Nestin is an intermediate filament found in nerve cells that has become widely used as a marker for neural precursors. Additionally, neurospheres are formed by different type of cells expressing also the SRY-box transcription factor-2 (Sox2) or the mRNA-binding protein Musashi-1 that has been demonstrated to play a critical role in promoting stem cell self-renewal [28]. The presence of these markers demonstrates that neurospheres from olfactory epithelium are formed by a heterogeneous population of cells in different stages of differentiation such as stem cells or proliferating neural progenitors [29].

**Fig. 3:**
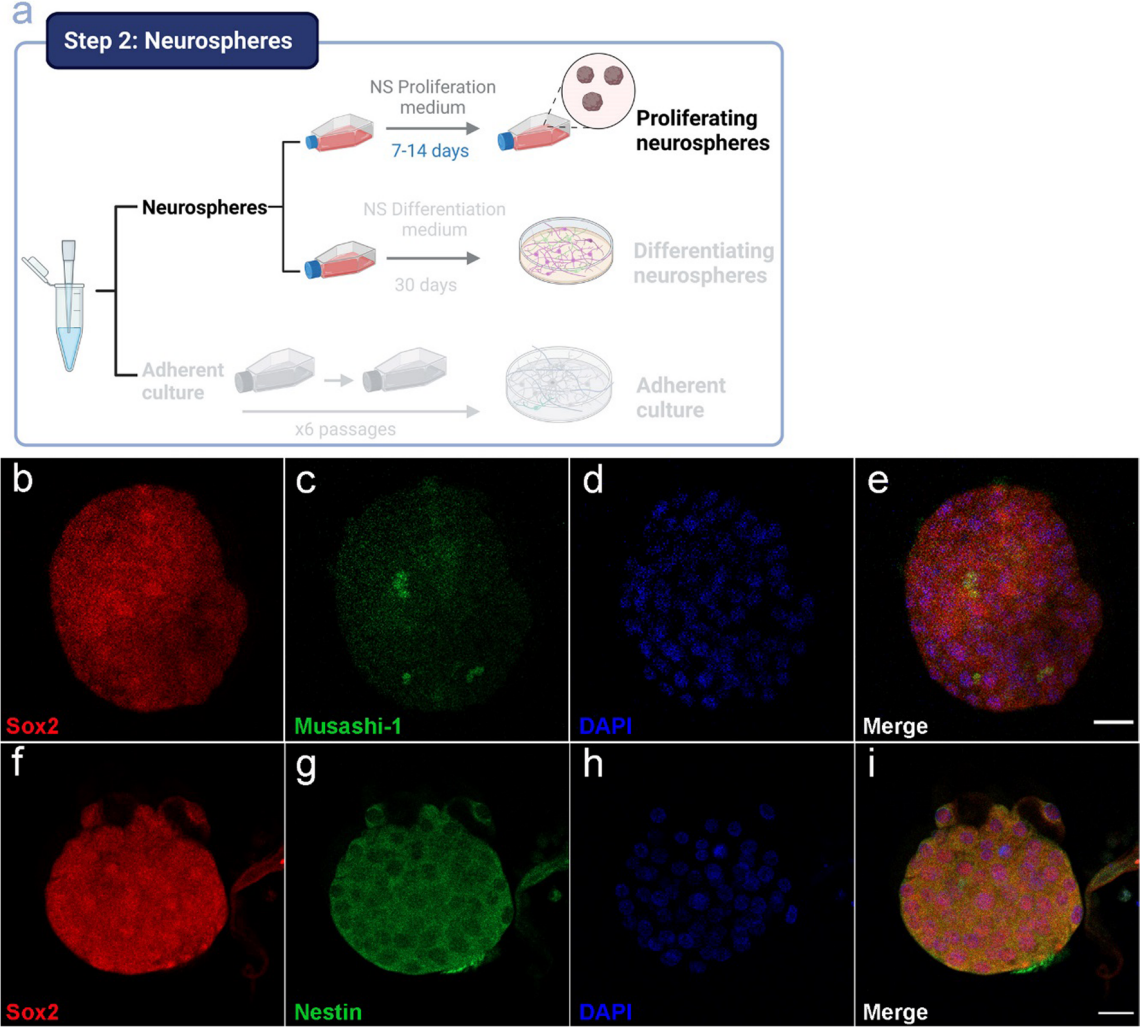
Characterization of proliferating neurospheres by immunocytochemistry. Schematic representation of the workflow for the culture of neurospheres (**a**). Immunostaining of neural markers Nestin, Sox2, and Musashi-1. Double labeling of Sox2 (**b**) and Musashi-1 (**c**) and merge image for Sox2 and Musashi-1 (**e**). Double labeling of Sox2 (**f**) and Nestin (**g**) and merge image for Sox2 and Nestin (**i**). DAPI was used to stain the nuclei (**d**, **h**). Bar = 20 µm

Characterization by immunocytochemistry (Fig. 3) using neural markers Sox2 (Fig. 3b, e, f, i), Musashi-1 (Fig. 3c, e), and Nestin (Fig. 3g, i) showed the co-expression of all these markers in the neurosphere culture. These results demonstrate that under our experimental conditions, cells from the olfactory epithelium are able to form neurospheres composed by neural progenitor cells, as previously demonstrated by other groups [7, 30–32].

### Olfactory Epithelium-Derived Neurospheres Give Rise to Differentiated Neural Progenitors

**Fig. 4:**
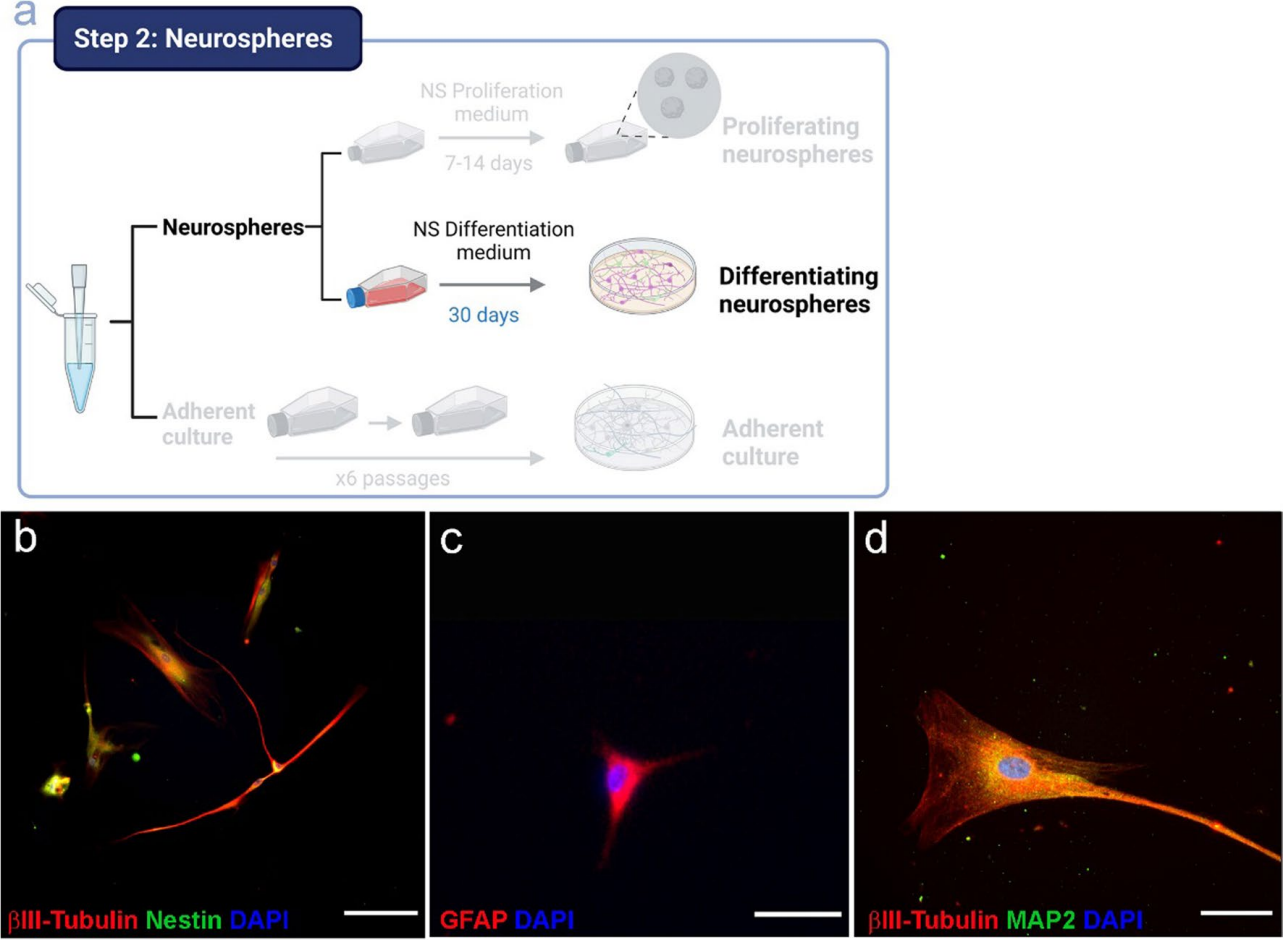
Characterization of differentiating cells from neurospheres. Schematic representation of the workflow for the culture of differentiating neurospheres (**a**). Immunostaining of βIII-Tubulin/Nestin (**b**), GFAP (**c**), and βIII-Tubulin/MAP2 (**d**). Nuclei were stained with DAPI. Bar = 200 µm (**b**, **c**) and 50 µm (**d**)

As described above, neurospheres are capable of giving rise to neurons and glial cells under the right conditions. When neurosphere culture medium was changed to differentiation medium (Fig. 4a), NPCs from the neurospheres begin to differentiate. Figure 4 shows cells expressing βIII-Tubulin (Fig. 4b, d), Nestin (Fig. 4b), GFAP (Fig. 4c), and MAP2 (Fig. 4d).

Despite being able to differentiate to cells expressing neuronal or glial markers, the number of cells obtained from neurospheres is extremely low. Moreover, when these culture of neurosphere-derived differentiated cells reaches confluence, we are not able to re-seed the culture and the cells die because mature neurons do not undergo cell division. As previously stated, the objective of the study was to obtain neuronal and glial cells for studying the cellular and molecular hallmarks of neuropsychiatric diseases. If the number of cells obtained is low, it would be difficult to use these cultures as a useful research tool. For this reason, we decided to grow adherent cultures from olfactory epithelium and then differentiate this culture to neurons and glia.

### Differentiation to Neuronal and Glial Cells from Adherent Cell Cultures

**Fig. 5:**
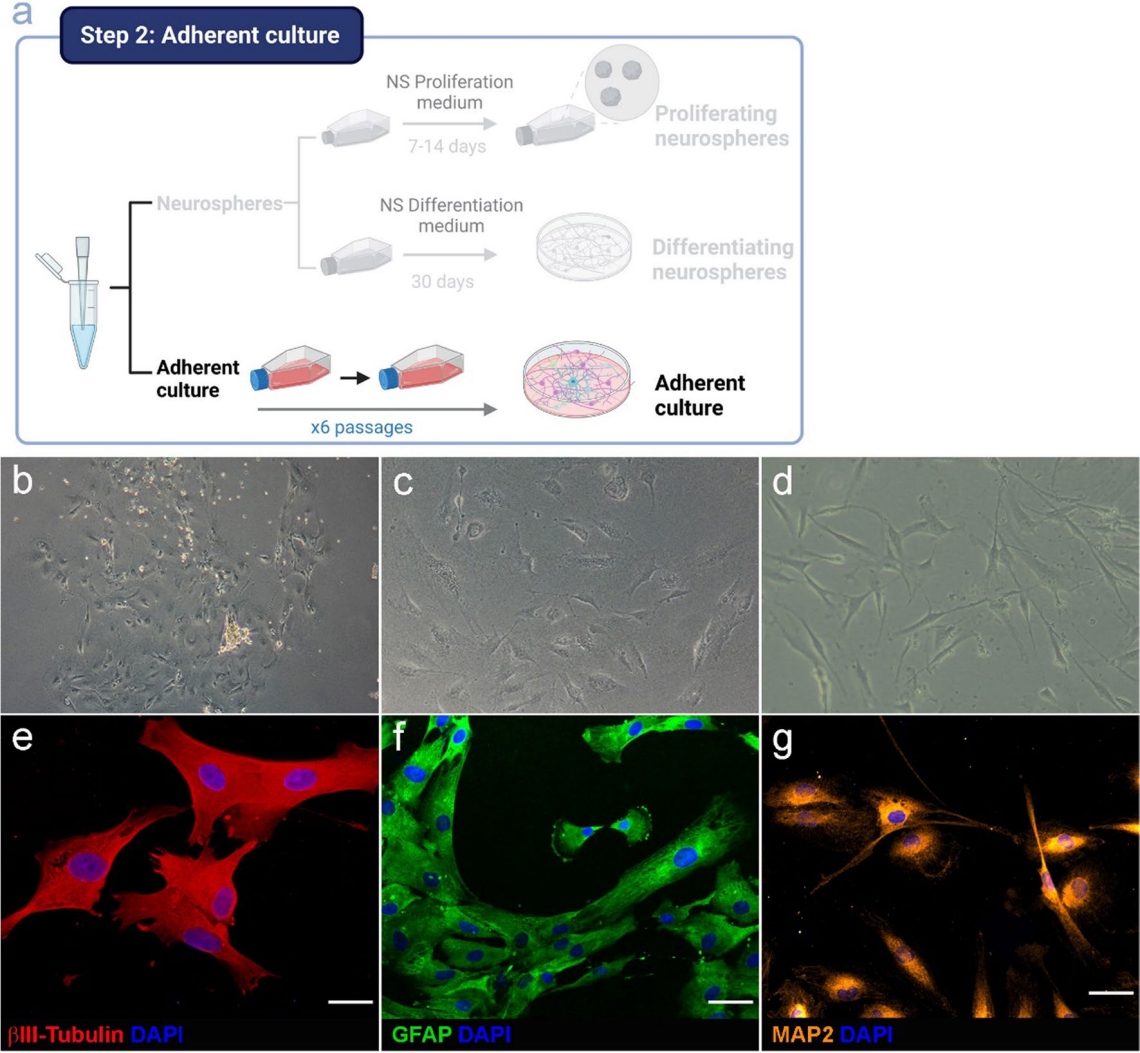
Characterization of adherent cells from olfactory epithelium. Upper panel shows the schematic representation of the workflow for the adherent cultures (**a**). Cells growing at different days after seeding (10X), at passage 0 (**b**), at passage 2 (**c**), and at passage 4 (**d**). Lower panel shows the immunocytochemistry of adherent cells on passage 5. Immunostaining of βIII-Tubulin (**e**), GFAP (**f**), and MAP2 (**g**). DAPI was used to stain the nuclei. Bar = 50 µm

As it has been previously explained, the number of differentiated cells obtained from neurospheres is extremely low for potentially being used in neuropsychiatric research. For this reason, the generation of neuron- or glia-enriched cultures was carried out from adherent cultures (Fig. 5a). As shown in Fig. 5, cells begin to grow after seeding (Fig. 5b, c, d). On passage 5, culture was mainly formed by a mix of cells at different stages of differentiation expressing neural markers such as βIII-Tubulin (Fig. 5e), GFAP (Fig. 5f), or MAP2 (Fig. 5g). These results indicate that under our specific conditions, cells from olfactory epithelium can be carried to a mix culture formed mainly by neural-fate cells and are in accordance with other previous studies that have reached this stage of culture for the study of neuropsychiatric disorders [6, 13, 16, 33, 34].

To this point, we aimed to separate this mix of cells in the adherent culture so that we could obtain neuronal- or glia-enriched different cultures. For this purpose, we tried to pick up from the adherent culture only the cells differentiating to neurons and seed these cells again in a new plate with a specific neuronal growth medium. For that, we carried out a magnetic labeling of the cells expressing PSA-NCAM in the surface. PSA-NCAM, the highly polysialated form of NCAM, is predominantly expressed in embryonic and neonatal neural tissue [35] and in proliferating late intermediate neuronal progenitor cells [36]. Indeed, PSA-NCAM persists in the adult brain in a spatially restricted way, contributing to synaptic plasticity, neuronal-glial structural remodeling, cell migration, and cell differentiation [37].

**Fig. 6:**
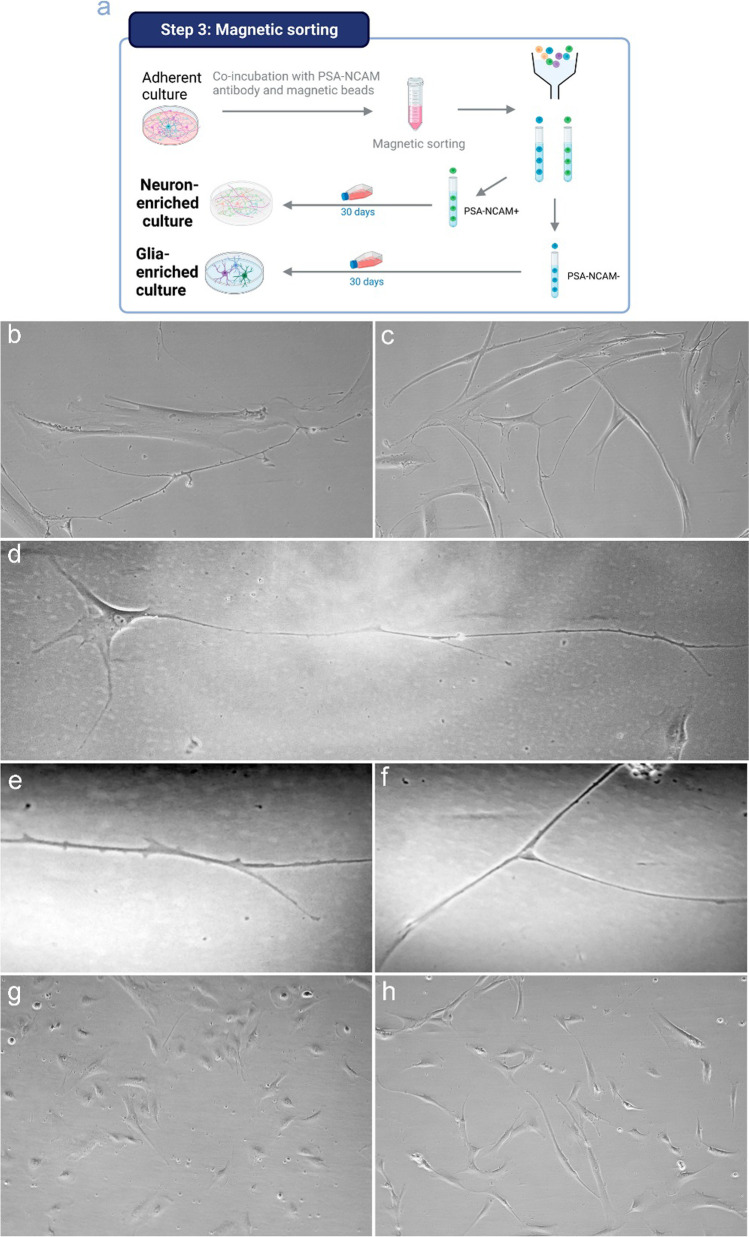
Bright-field visual characterization of the neuron- or glia-enriched cultures. Schematic representation of the workflow for the magnetic selection of cells (**a**). Bright-field image of PSA-NCAM-positive cells (neuron-enriched fraction) (10X) showing neuronal shape and long prolongations connecting with other cells (**b**, **c**). Differentiated cell with characteristic neuron shape (10X) (**d**). Prolongation of a neuron (20X) (**e**) and end of prolongation of a neuron in a synaptic button-like connection between two different cells (20X) (**f**). Bright-field image of PSA-NCAM-negative cells (glia-enriched fraction) (10X) showing a different growth pattern, with larger aggregates and less elongated cells (**g**, **h**)

After magnetic labeling, cells were separated by magnetic activated cell sorting, and both fractions, labeled (PSA-NCAM^+^, neuronal-enriched fraction) and non-labeled (PSA-NCAM^−^, glia-enriched fraction), were completely recovered and cultured in differentiation medium. Figure 6 shows the morphological shape of both types of cultures after 30 days of culture. PSA-NCAM^+^ cells, with a higher proportion of neurons, show a more neuronal morphology, with more elongated shapes (Fig. 6b, c). Moreover, we found differentiated cells with a characteristic neuron shape (Fig. 6d, e, f). In contrast, cultures with a higher proportion of glial cells or lower proportion of neuronal cells (PSA-NCAM^−^ cultures), show a completely different growth, with larger cell aggregates and less elongated cells (Fig. 6g, h).

### Neuron-Enriched or Glia-Enriched Cultures Express-Specific Markers of Their Cellular Fate

**Fig. 7:**
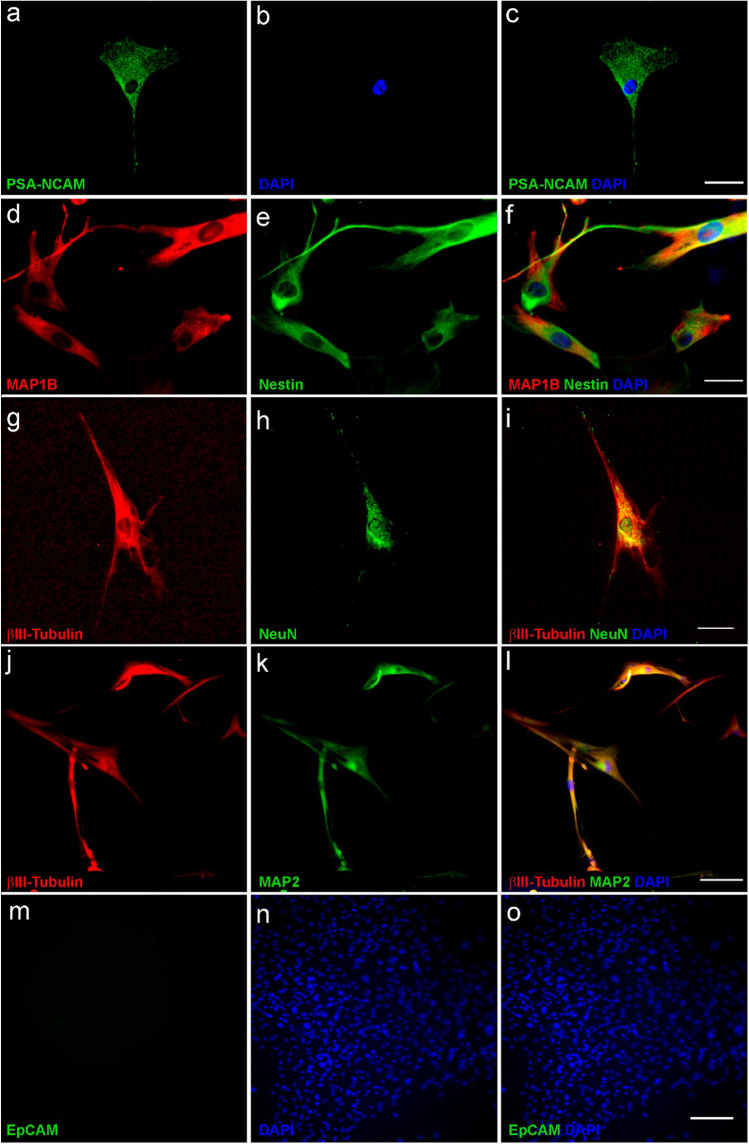
Neuron-enriched cultures express specific neuronal markers. Immunocytochemistry of neuron-enriched culture derived from the olfactory epithelium. Immunostaining of PSA-NCAM (**a**, **c**). Double labeling of MAP1B (**d**) and Nestin (**e**) and merge image for MAP1B and Nestin (**f**). Double labeling of βIII-Tubulin (**g**) and NeuN (**h**) and merge image for βIII-Tubulin and NeuN (**i**). Double labeling of βIII-Tubulin (**j**) and MAP2 (**k**) and merge image for βIII-Tubulin and MAP2 (**l**). Labeling of EpCAM (**m**, **o**). DAPI was used to stain the nuclei (**b**, **n**). Bar = 200 µm

After the magnetic separation, approximately 5 days are needed for the culture to stabilize; the cells that survive the magnetic separation begin to adhere to the flask and to differentiate. At this time, we perform the PSA-NCAM immunocytochemistry to ensure the quality of the separation (Fig. 7a, b, c). Afterwards, we let the cultures grow for approximately 30 days. Upon reaching 30 days, the cultures have a sufficient number of cells in each flask (F75) in both types of cultures. These 30-day F75s are the ones that we will use to carry out the different experiments in the patients. For the characterization of both types of cultures, the expression of specific neural and glial markers was evaluated by immunocytochemistry. As seen in Fig. 7, neuron-enriched cultures abundantly express neuronal proteins, such as MAP1B (Fig. 7d). This protein is expressed predominantly during the early stages of development of the nervous system, where it regulates processes such as axonal guidance and elongation. In the adult brain, it participates in the regulation of the structure and physiology of dendritic spines in glutamatergic synapses and is also found in presynaptic synaptosomal preparations [38]. Nestin (Fig. 7e) is a class VI intermediate filament protein and a specific marker of neural stem/progenitor cells [39] that can differentiate into neurons and glial cells. Co-expression of both proteins was found in the neuronal-enriched cultures (Fig. 7f). These cultures also express βIII-Tubulin (Fig. 7g, j) together with NeuN (Fig. 7h) and with MAP2 (Fig. 7k) as demonstrated the immunocytochemistry assays. Moreover, co-expression of these proteins was found in the neuronal-enriched cultures (Fig. 7i, l).

Tubulin is a major constituent protein of microtubules and consists of two 50-kDa subunits designated as α and β. Expression of class III β-Tubulin, one of the six isotypes of the β subunit expressed in mammals [40], is restricted almost entirely to neurons [41]. In addition, NeuN is a well-recognized marker that is detected in post-mitotic neurons being distributed in the nuclei of neurons in almost all parts of the nervous system and is stably expressing during specific stages of development [42]. The co-expression of these two neuronal markers has been reported in differentiating and mature neuronal cells from diverse sources [43–45]. For this reason, the co-expression of NeuN and βIII-Tubulin in the neuron-enriched cultures suggests that these cultures are formed by a mix of neurons at different stages of differentiation.

On the other hand, MAP2 is the predominant cytoskeletal regulator within neuronal dendrites, and a robust somatodendritic marker due to its abundance and specificity. It influences microtubule dynamics and microtubule/actin interactions to control neurite outgrowth and synaptic functions [46].

A limitation of the study is the use of magnetic beads with PSA-NCAM since this protein is a marker for immature neuronal-committed progenitors, which again, due to the immaturity of the cells, can make the selection less effective. Nevertheless, to date, PSA-NCAM is the only neuron-specific protein expressed in the surface of the cell that can be used for the magnetic sorting in humans. In addition, for discarding the presence of epithelial cells in this culture of ON-derived cells, we carried out an immunocytochemistry assay for the labeling of EpCAM, which was not found in the culture (Fig. 7m, o).

These results suggest that the vast majority of cells in the neuron-enriched culture are neurons at different stages of development, with null presence of epithelial cells.

**Fig. 8:**
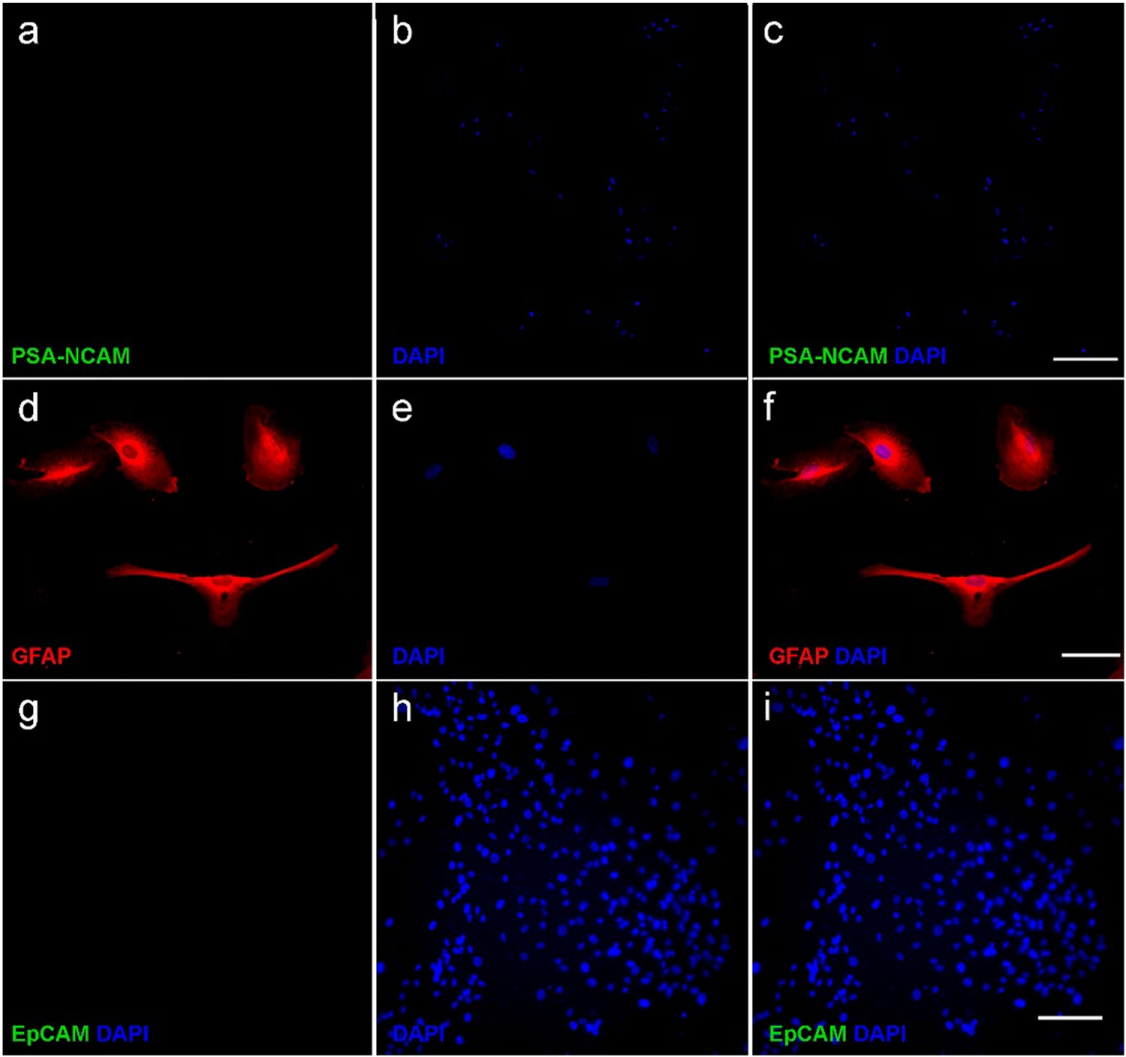
Glia-enriched cultures express specific glial markers. Immunocytochemistry of glia-enriched culture derived from the olfactory epithelium. Immunostaining of PSA-NCAM (**a**, **c**). Labeling of GFAP (**d**, **f**) and EpCAM (**g**, **i**). DAPI was used to stain the nuclei (**b**, **e**, **h**). Bar = 200 µm

In addition, we did not find the presence of PSA-NCAM (Fig. 8a, b, c) in the glia-enriched cultures immediately after the magnetic separation. Moreover, in glia-enriched cultures, as seen in Fig. 8, immunocytochemistry assays demonstrated the presence of specific glial proteins such as GFAP (Fig. 8d, e, f), but not the epithelial EpCAM (Fig. 8g, h, i).

To confirm these results and further characterize the two cell-type populations, we performed a flow cytometry analysis by labeling the cells either with NeuN or GFAP. We use the NeuN marker to demonstrate that in these cultures, 30 days after the magnetic separation, there is a sufficient amount of mature neurons in the culture, although the culture may potentially present neurons at different stages of differentiation, which demonstrates the presence of markers of immature neurons in the immunocytochemical characterization as previously explained.

**Fig. 9:**
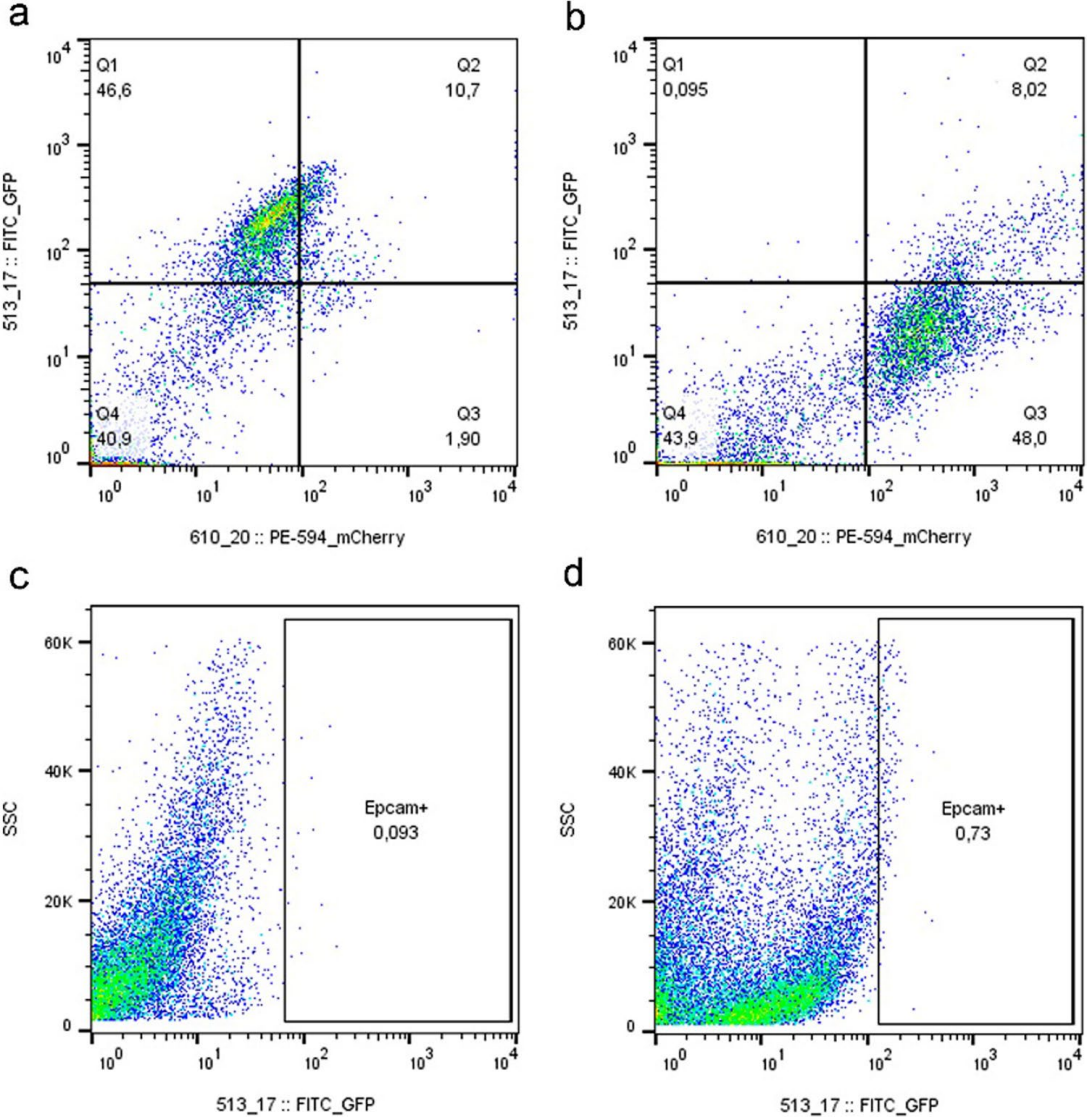
Fluorescence-activated cell sorting (FACS) of neuron-enriched or glia-enriched cultures. The purity of enriched cultures was determined by performing post-sort FACS analysis on samples of neuron or glia cell subpopulations and assessing the percentages of cells in each gate. Sorting cells under the described conditions yielded over > 46% NeuN- and FITC-positive cell populations in neuron-enriched cultures (**a**) and 48% GFAP- and mCherry-positive cell populations in glia-enriched cultures (**b**). FACS plots of negative control for EpCAM in neuron-enriched cultures (**c**) and in glia-enriched cultures (**d**) are displayed in the lower panel

Besides, we also labeled the cultures with EpCAM to discard the presence of epithelial cells. As seen in Fig. 9, we detected an increase of up to 46% of NeuN-positive cell population in the culture of cells positively selected with PSA-NCAM (Fig. 9a). In contrast, the flow cytometry analysis only gave a residual value of 1.90% of GFAP-positive cells in this neuronal-enriched culture.

The percentages obtained on FACS may be so low because the cells suffer during sample processing and may be dying. Although it is difficult to be sure without a propidium iodide (PI) or other viability marker analysis, this population negative to both markers may correspond to cells that are dying due to sample processing since looking at their appearance in forward scatter (FSC) vs. side scatter (SSC), all cells are very stuck together and in values close to 0.

In the glia-enriched fraction, an increase of up to 48% of GFAP-positive cell population was found in the culture of cells negatively selected with PSA-NCAM (Fig. 9b). In contrast, the flow cytometry analysis only gave a residual value of 0.095% of NeuN-positive cells. Moreover, the flow cytometry analysis gave a residual value of 0.093% of EpCAM-positive cells in neuronal-enriched cultures (Fig. 9c) and 0.73% in glia-enriched cultures (Fig. 9d).

These results demonstrate that our methodology has improved the purity of the cultures, obtaining subpopulations of neurons or glia at different stages of development.

### Different Pattern of Measurable Molecules in Neuron-Enriched and Glia-Enriched cultures

As previously explained, the final objective of the work is to obtain a biological substrate of the patient in which we will be able to find and measure trait, state, or prognostic biomarkers of the disease. For this reason, we wanted to know if these enriched cultures may produce and release molecules that we could measure in the culture medium and that could serve as potential biomarkers. To do this, we decided to measure some biomarkers already established in psychiatric research, which are related to the inflammatory component of the disease, such as IFNγ, PGE2, IL-6, or the Kyn/Trp ratio [47].

**Fig. 10:**
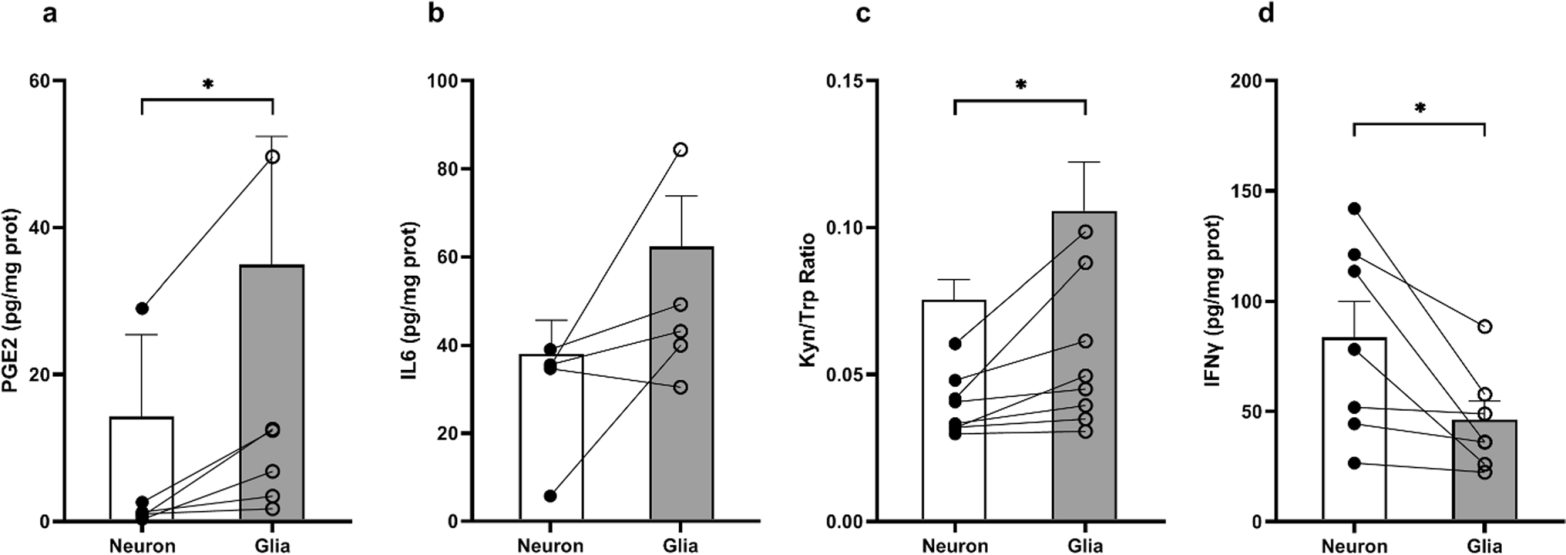
Levels of PGE2, IL6, Kyn/Trp, and IFNγ in the medium of neuron-enriched or glia-enriched cultures. Levels of PGE2 (**a**), IL6 (**b**), ratio of Kyn/Trp (**c**), and IFNγ (**d**) measured by ELISA assay in both neuron-enriched and glia-enriched culture medium and expressed in pg/mg protein. Bars represent the mean, and circles represent individual values (*n* = 5–8 subjects). Statistical analyses consisted on a paired *T*-test for each subject comparing the levels of the different molecules in neuron or glial cultures. Significance was set at.^*^p < 0.05

As seen in Fig. 10, both types of cultures are capable to produce and release these compounds. Moreover, significant differences were found in the levels of PGE2 (Fig. 10a), Kyn/Trp ratio (Fig. 10c), and IFNγ (Fig. 10d) between neuron- and glia-enriched cultures, while no significant changes were found in the IL6 levels (Fig. 10b). Specifically, levels of PGE2 and kynunerine were significantly increased in glia-enriched cultures compared to neuron-enriched cultures, while IFNγ levels were higher in neuronal than in glia-enriched cultures. These results demonstrate that both types of cultures exhibit different levels of these measurable molecules in the culture medium.

## Limitations and Concluding Remarks

To our knowledge, this is the first study describing a method for the generation of sufficient amount of human neurons or glial cells subpopulations in living subjects in an easy and non-invasively form. Other studies had developed similar methods but obtaining the sample invasively in biopsies after nasal surgery and with anesthesia, or even postmortem in autopsies. On the other hand, other groups have developed and characterized non-invasively obtained adherent cultures of the olfactory epithelium in patients, but none of them has reached the step of selecting and culturing neurons and glia.

The study has some limitations that obviously cannot be ignored. First, what we have obtained after selecting the cells based on the presence or absence of PSA-NCAM are cultures enriched in neurons or glia, but not pure cultures. Another limitation is the fact that these neuron and glial cells have developed in vitro, without the natural inputs that they would have had if they had developed physiologically. It must be further studied whether the manipulation of neural progenitors has interfered in their development in terms of the ability to reproduce some alterations inherent to psychiatric or neurological conditions. Moreover, these cultures may be considered with an “olfactory-specific” origin which should be kept in consideration when comparing with other models.

Despite these limitations, we believe that this work has an important clinical impact since it will allow the characterization of disease fingerprints through the study of neurons and glial cells from patients.

## Supplementary Information

Below is the link to the electronic supplementary material.

Supplementary file1 (DOCX 14 KB)

Supplementary file2 (DOCX 13 KB)

## Data Availability

The datasets generated during and/or analyzed during the current study are available from the corresponding author on reasonable request.
