# Supplementary material for: Isolation and Differentiation of Neurons and Glial Cells from Olfactory Epithelium in Living Subjects
Source: Mol Neurobiol. 2023 Apr 28;60(8):4472–87. doi: 10.1007/s12035-023-03363-2 (PMC10293402; doi:10.1007/s12035-023-03363-2)
Supplement: Supplementary file 1 — Supplementary file1 (DOCX 14 KB) [file 12035_2023_3363_MOESM1_ESM.docx]

**Table 1. Primary antibodies**

| **Antibody** | **Host** | **Company, Catalog #** | **Concentration** |
| --- | --- | --- | --- |
| $\beta$III-Tubulin | rabbit | StemCell, 60052 | 1:1000 |
| EpCAM | mouse | ThermoFisher, 11-5791-82 | 1:500 |
| GFAP | rabbit | DAKO, Z0334 | 1:4000 |
| MAP1B | rabbit | ThermoFisher, PA5-82798 | 1:500 |
| MAP2 | mouse | ThermoFisher, 13-1500 | 1:500 |
| Musashi-1 | rat | Thermofisher, 14989682 | 1:500 |
| Nestin | mouse | Merck, MAB5326 | 1:500 |
| NeuN | mouse | Merck, MAB377 | 1:500 |
| PSA-NCAM | mouse | Miltenyi, 130-117-394 | 1:1000 |
| Sox2 | rabbit | ThermoFisher, PA1094 | 1:1000 |

Primary antibodies, host, company and concentration used for immunofluorescence assays
