# Supplementary material for: Isolation and Differentiation of Neurons and Glial Cells from Olfactory Epithelium in Living Subjects
Source: Mol Neurobiol. 2023 Apr 28;60(8):4472–87. doi: 10.1007/s12035-023-03363-2 (PMC10293402; doi:10.1007/s12035-023-03363-2)
Supplement: Supplementary file 2 — Supplementary file2 (DOCX 13 KB) [file 12035_2023_3363_MOESM2_ESM.docx]

**Table 2. Secondary antibodies**

| **Antibody** | **Host** | **Company, Catalog #** | **Concentration** |
| --- | --- | --- | --- |
| Alexa Fluor™ 594 | goat anti-rabbit | ThermoFisher, A-11037 | 1:1000 |
| Alexa Fluor™ 488 | goat anti-rat | ThermoFisher, A-11006 | 1:1000 |
| Alexa Fluor™ 488 | donkey anti-mouse | ThermoFisher, A-21202 | 1:1000 |

Secondary antibodies, host, company and concentration used for immunofluorescence assays
